# Relationship between dental follicle, gubernacular canal and developmental theories in supernumerary teeth from different regions of the jaws

**DOI:** 10.1007/s11282-026-00920-y

**Published:** 2026-04-08

**Authors:** Julia Beatriz Tonon, Henrique Mateus Alves Felizardo, Aline Cristina Tonon-Alves, Hugo Gaêta-Araujo

**Affiliations:** 1 Department of Stomatology, Public Health, and Forensic Dentistry, Ribeirão Preto School of Dentistry, University of São Paulo (USP), Avenida do Café, s/n - Monte Alegre, Ribeirão Preto, São Paulo 14040-904 Brazil; 2 Department of Dental Materials and Prosthesis, Ribeirão Preto School of Dentistry, University of São Paulo (USP), Avenida do Café, s/n - Monte Alegre, Ribeirão Preto, São Paulo 14040-904 Brazil; 3 Independent Statistician, R. General Osório, 1180 – Velha, Blumenau, Santa Catarina 89041- 002 Brazil

**Keywords:** Unerupted tooth, Supernumerary tooth, Tooth eruption, Tooth germ, Odontogenesis

## Abstract

**Objectives:**

Evaluate the characteristics of dental follicle and gubernacular canal (GC) in supernumerary teeth from different regions of dental arches, using CBCT scans of non-syndromic patients, and associate with developmental theories.

**Materials and methods:**

Sample was composed of CBCT scans of 408 intraosseous supernumerary teeth. The characteristics of the supernumerary teeth, the relationship of their follicular space with adjacent teeth, and the presence or absence of the GC were assessed. Chi-square tests were used to compare categorical variables among supernumerary teeth from different regions, with a significance level of 5%.

**Results:**

Isolated follicles were more frequent in the maxillary molar region (77.3%), follicles with contact relationships in the mandibular incisor region (42.9%), and fused follicles in the maxillary incisor region (25.8%). Furthermore, the detection rate of the GC varied from 15% to 59%, depending on the region of the dental arch. Most incisors were associated with the theory of complete dichotomy. In the premolar region, the most frequent theory was that of dental lamina hyperactivity.

**Conclusion:**

The relationship between follicle characteristics and the GC underlies the formulation of hypotheses regarding different development theories of supernumerary teeth. Thus, we can hypothesize that supernumerary teeth in the premolar region most commonly arise from dental lamina hyperactivity, whereas in other regions, the dichotomy theory may best explain their development, especially in the maxilla.

**Clinical Relevance:**

Understanding GC patterns in supernumerary teeth may help clinicians anticipate tooth behavior and its relationship with adjacent teeth, leading to more accurate diagnosis and surgical planning.

## Introduction

The gubernacular cord is a structure composed of connective tissue interspersed with epithelial islands that connect the dental follicle to the overlying gingiva and functions to guide or direct the course of tooth eruption [1]. Its formation begins from remnants of the dental lamina, which organize into a fibrous cord, extending the reduced enamel epithelium of the enamel organ toward the oral mucosa. This structure is in the alveolar ridge, behind the deciduous tooth [2]. Bone resorption around this cord leads to the formation of the gubernacular canal [1].

Both the gubernacular cord and its canal appear to play an important role in tooth eruption, guiding the developing tooth toward the alveolar process [3, 4]. Consequently, several recent studies have reported the characteristics of the gubernacular canal as visualized by cone-beam computed tomography (CBCT). The absence of the canal or alterations in its location within the dental follicle are associated with teeth exhibiting abnormal eruption processes [4]. Furthermore, previous studies have shown associations between the gubernacular canal and pathological processes such as odontogenic cysts and tumors [5], as well as odontomas [6, 7]. Beyond the normal teeth, some studies have investigated the characteristics of the gubernacular canal in supernumerary teeth. Supernumerary teeth are teeth or tooth-like structures that may erupt or remain impacted, in addition to the 20 deciduous and 32 permanent teeth of the normal dentition [8].

In the study by Kaplan et al., 2020 [9], 63 impacted supernumerary teeth from 44 patients were evaluated using CBCT scans. The study reported a gubernacular canal frequency of 31.7%, with no significant differences in the frequency or characteristics of the canal between sexes, ages, quadrants, or tooth positions. However, the authors noted that studies in larger populations are still needed to obtain more detailed information. Similarly, Zengin et al., 2023 [10] reported a detection rate of 77.2% in 144 impacted supernumerary teeth, with variations in canal morphology and direction, although without statistically significant differences. Both studies highlight sample size limitations.

In a recent study, Nishina et al., 2024 [3] evaluated the presentation of the gubernacular canal in supernumerary teeth of nine syndromic patients with cleidocranial dysplasia. A total of 97 teeth were assessed, including 59 supernumerary teeth. The most relevant finding was that in 44 cases, two or more teeth shared a single dental follicle. Of these teeth, 79.5% had only one gubernacular canal, while in 20.5% of cases, the canal was not detected. The authors suggested that this specific configuration of dental follicles with a single gubernacular canal may indicate that two or three teeth develop from a single dental lamina during tooth development in patients with cleidocranial dysplasia.

Several theories have been proposed to explain the etiology of supernumerary teeth, including genetic and environmental factors [8]. The main developmental theories of supernumerary teeth include the dental lamina hyperactivity theory, the dichotomy theory, and the progression zone theory. In brief, the dental lamina hyperactivity theory proposes excessive proliferation of dental lamina leading to additional tooth germs. The dichotomy theory suggests splitting of the tooth germ, resulting in supernumerary formation. The progression zone theory relates to abnormal proliferation in specific regions of the dental arch. However, evidence correlating these theories with tooth location remains limited [8].

With the growing use of CBCT, the gubernacular canal has gained attention due to its higher detection in these scans. It is now understood that the gubernacular canal is directly involved in tooth eruption, acting as a guide. Importantly, its origin from dental lamina remnants links this structure to the developmental mechanisms of supernumerary teeth. Although previous studies have evaluated the gubernacular canal in supernumerary teeth, small sample size has been reported as a limitation and those did not associate the characteristics of the gubernacular canal with developmental theories. Furthermore, there is a lack in scientific literature concerning the association between the presence and characteristics of the gubernacular canal with supernumerary teeth in different regions of the jaws. These findings may provide evidence to explain potential developmental theories of supernumerary teeth. In this scenario, our hypotheses are based on the findings of Nishina et al., 2024 [3], regarding the presence of dental follicles containing two or more teeth, with or without a gubernacular canal. Accordingly, four possible conditions could be observed: (1) a single tooth in a dental follicle without a gubernacular canal; (2) a single tooth in a dental follicle with a gubernacular canal; (3) two or more teeth in a dental follicle without a gubernacular canal; and (4) two or more teeth in a dental follicle with a gubernacular canal. Condition 1 could be explained by follicular total dichotomy, condition 2 could be related to dental lamina hyperactivity, while conditions 3 and 4 could correspond to partial dichotomy or dental lamina hyperactivity. Additionally, we hypothesize that each of these dental follicle/gubernacular canal characteristics may be associated with the different regions in which supernumerary teeth develop.

Thus, the objective of the present study is to evaluate the characteristics of the dental follicle, as well as the detection rate of the gubernacular canal in supernumerary teeth from different regions of the dental arches, using CBCT scans of non-syndromic patients, to gather evidence supporting the developmental theories of supernumerary teeth.

## Materials and methods

This was a retrospective cross-sectional study conducted at the Laboratory for Innovation in Oral Radiology and Imaging (LIRIO), within the Department of Stomatology, Public Health, and Forensic Dentistry (DESCOL). The present research was approved by the Research Ethics Committee of the Ribeirão Preto School of Dentistry (FORP-USP), under protocol number 6.980.465 (CAAE 80541124.5.0000.5419).

The null hypothesis of the study is that there is no difference in dental follicle characteristics and detection rate of the gubernacular canal across different regions of the dental arches. The primary outcome of the study is the absolute and relative frequency of dental follicle characteristics and the detection rate of the gubernacular canal in supernumerary teeth from different regions of the dental arches.

### Sample size calculation

The sample size calculation was based on the study by Nishina et al., 2024 [3], considering the proportions found for the detection of the gubernacular canal in supernumerary teeth that either shared or did not share a dental follicle with a normal-count tooth (79.5% vs. 86.8%). The calculation was performed using the “pwr.2p.test” function in R, which determines the parameters necessary to achieve a desired statistical power. The effect size was set at 0.2, with a significance level of 5% and a test power of 80% for a two-tailed test, resulting in a sample of 408 supernumerary teeth.

### Sample selection

CBCT images were obtained from the image database of the Radiology Clinic at FORP-USP. Accordingly, the sample comprised CBCT scans of patients presenting intraosseous supernumerary teeth that met the inclusion and exclusion criteria described below. For sample selection, the Radiology and Radioprotection Section (SCRAD) of FORP-USP was requested to select CBCT scans of patients referred to the Oral Radiology service for evaluation of supernumerary teeth, encompassing all ages. The CBCT scans of the database were acquired using two CBCT devices, OP300 (Instrumentarium, Tuusula, Finland) and Eagle 3D (Dabi Atlante, Ribeirão Preto, Brazil). Both devices operated under variable acquisition protocols of kilovoltage (85 or 90 kV), tube current (2 to 13 mA), voxel size (85 to 320 μm), and field of view (FOV 5 × 5 to 23 × 16 cm). The scans were exported by SCRAD radiology technicians, who were not part of the research team, using an image acquisition software (Cliniview, Instrumentarium, Tuusula, Finland) with data anonymization. The only accompanying data were a random number corresponding to each scan, and the patient’s sex and age, ensuring that patient identification was impossible and maintaining confidentiality and privacy. All scans were exported in DICOM format and stored on an external hard drive.

After the initial selection by SCRAD, the final sample was determined by applying the following inclusion and exclusion criteria.

### Inclusion criteria

CBCT scans showing intraosseous supernumerary teeth in formation, in the process of eruption, or impacted were included.

### Exclusion criteria

Teeth in advanced eruption stages, where the follicular space is so close to the alveolar ridge that detection of the gubernacular canal is impossible. Fully erupted supernumerary teeth were excluded, as the evaluation of the gubernacular canal requires the presence of an intraosseous follicular space. The scans with motion artifacts preventing proper evaluation, and syndromic patients with systemic alterations affecting bone were excluded.

### Sample evaluation

For each scan, the patient’s sex and age were recorded, along with the dental group of the supernumerary teeth according to the dental arch (maxilla or mandible) and their region (incisors, canines, premolars, or molars). Once classified, the teeth were evaluated as follows [adapted from Gaêta-Araujo et al., 2019 [4]:

*Stage of development*: crypt (no crown calcification visible), crown (from the beginning of enamel formation to the cement-enamel junction), up to half of the root (from the beginning of root formation until the root reaches the same length as the crown), beyond half of the root (root length exceeding crown length until the apex remains open), closed apex (tooth fully formed), or indeterminate (when extreme alterations in morphology did not allow assessment of the stage of development).

*Morphology*: indeterminate (when in crypt stage, morphology cannot be identified), normal (corresponding to teeth of that region), altered (microdontia, macrodontia, or other shape anomalies), or compound odontoma (three or more denticles within the same dental follicle).

*Tooth inclination*: indeterminate (when in crypt stage, inclination cannot be determined), vertical (long axis parallel to the long axis of adjacent teeth), inclined (long axis angled relative to adjacent teeth), horizontal (long axis perpendicular to adjacent teeth), or inverted (long axis exceeding 90° relative to adjacent teeth).

*Relationship between the supernumerary tooth follicle and adjacent intraosseous teeth*: isolated (follicle boundaries can be determined and no proximity to adjacent tooth follicles), in contact (follicle boundaries can be determined but touching adjacent tooth follicles), or fused (contact with adjacent tooth follicles with indistinguishable boundaries in the contact region).

*Number of adjacent teeth*: for teeth in contact or fused with adjacent teeth, the number of adjacent teeth in these conditions was recorded.

*Type of adjacent teeth*: for teeth in contact or fused with adjacent teeth, they were categorized as normal-count teeth, supernumerary teeth, or both.

*Gubernacular canal*: absent or present (when a hypodense band originating from the dental follicle and extending toward a bone surface was identified).

The selected scans were then evaluated using OnDemand3D software (Cybermed Inc., Seoul, Republic of Korea) on an MDRC-22 medical imaging monitor (BARCO, Kortrijk, Belgium), in a quiet environment with low ambient lighting suitable for image analysis. The evaluation was performed dynamically, navigating through all slices corresponding to the tooth under analysis. To begin the tooth assessment, the tomographic slices were aligned with the long axis of the tooth in the axial, sagittal, and coronal planes.

Initially, 200 cases were evaluated by two examiners, both dentists with experience in oral radiology (CBCT interpretation). After individual evaluations, the findings were verified jointly by both evaluators. Subsequently, 100 cases were analyzed by an oral and maxillofacial radiologist with more than 10 years of CBCT analysis experience and compared with the previous evaluations to refine calibration. Once these two calibration stages were completed, the entire sample was evaluated by a single examiner, including re-evaluation of the initial 200 cases.

### Relationship between the dental follicle, gubernacular canal, and developmental theories

**Fig. 1:**
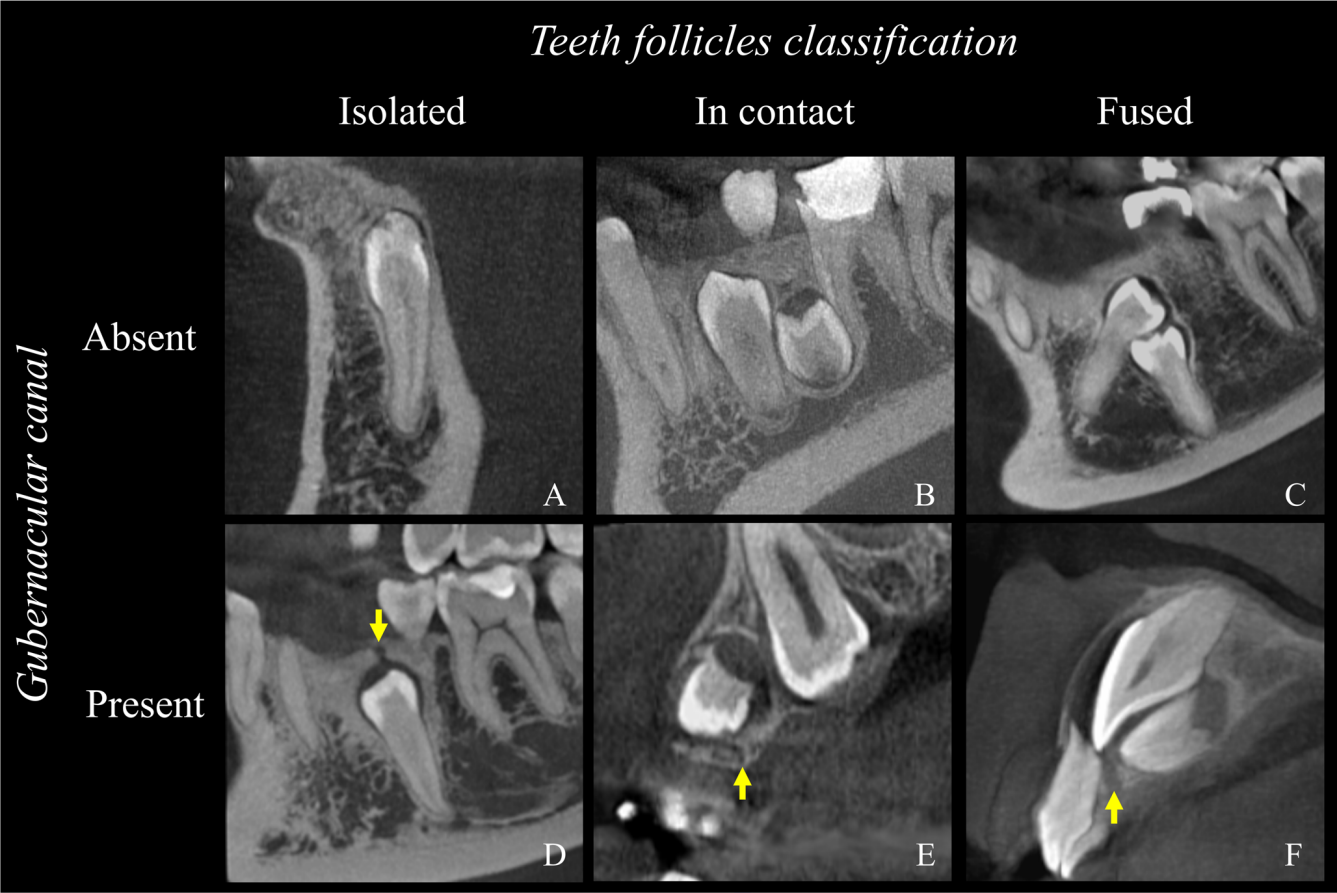
CBCT images with the classification of tooth follicles considering their relationship with adjacent teeth (isolated, in contact or fused) and the presence (indicated by the yellow arrows) or absence of the gubernacular canal. **A** Supernumerary tooth in lower canine region with an isolated dental follicle and absence of the gubernacular canal;** B** Supernumerary tooth in lower premolar region with the follicle in contact with the adjacent tooth and absence of the gubernacular canal. **C** Supernumerary tooth in lower premolars region with fusion between the dental follicles of adjacent teeth and absence of the gubernacular canal.** D** Supernumerary tooth in lower premolars region with an isolated follicle and presence of the gubernacular canal.** E** Supernumerary tooth in upper canine region with the follicle in contact with the adjacent tooth and presence of the gubernacular canal.** F** Supernumerary tooth in upper central incisor region with fusion between the dental follicles of adjacent teeth and presence of the gubernacular canal

Based on the analysis of the relationship between the supernumerary tooth follicle and adjacent intraosseous tooth follicles, along with the presence or absence of the gubernacular canal (Fig. 1), hypotheses were formulated regarding the main developmental theories of supernumerary teeth: the dichotomy theory and the dental lamina hyperactivity theory. These hypotheses consider that teeth presenting a gubernacular canal originate from dental lamina and thus suggests development according to the hyperactivity theory. Conversely, the absence of the gubernacular canal may be suggestive of the follicular dichotomy. Accordingly, teeth with isolated follicles without a gubernacular canal suggest complete dichotomy, whereas teeth with isolated follicles and with a gubernacular canal could suggest the dental lamina hyperactivity theory. Supernumerary teeth with follicles in contact but lacking a gubernacular canal are suggestive of partial dichotomy, while teeth with follicles in contact and a gubernacular canal suggest dental lamina hyperactivity.

Regarding fused follicles, those without a gubernacular canal could be related to partial dichotomy, whereas fused follicles with a gubernacular canal could originate from dental lamina hyperactivity if the canal within the follicle belonged to that tooth, or from partial dichotomy if the canal belonged to another tooth. To determine which tooth in a fused follicle the gubernacular canal belonged to, the tooth type (normal-count or supernumerary) and its proximity to the canal were taken into account (in cases with more than one supernumerary tooth).

### Data analysis

Data analysis was performed using SPSS version 25.0 (IBM SPSS Statistics, Armonk, NY, USA) and Prism version 8.0 (GraphPad, La Jolla, CA, USA), with a significance level set at 5%. The chi-square test was used to compare tooth characteristics (region, development stage, morphology, and angulation) between dental arches, as well as to evaluate differences in dental follicle characteristics and gubernacular canal detection rates across regions of the dental arches. Additionally, it was applied to assess the association between the presence of the gubernacular canal and dental follicle characteristics across regions and dental arches, including their possible relationship with developmental theories.

## Results

A total of 408 supernumerary teeth were evaluated, and their characteristics are shown in Table 1. There were statistically significant differences for all characteristics (region, formation, morphology, and inclination) comparing teeth from the maxilla and the mandible (*p* < 0.05). Of the 408 teeth evaluated, 206 (50.5%) were in the maxilla and 202 (49.5%) in the mandible, as shown in Table 1. In the maxilla, supernumerary teeth were predominant in the incisor region (45.1%), while in the mandible it was in the premolar region (80.2%). Regarding the stage of development (Table 1), fully formed teeth were more prevalent in the maxilla (38.3%) compared to the mandible (26.7%). Concerning morphology, altered morphology was more common in maxillary teeth (57.8%), while normal morphology was more common in the mandible (76.2%). Finally, regarding inclination, both maxillary and mandibular teeth presented similar rates of vertical teeth (48.1 and 55.0%, respectively), although maxillary teeth were more commonly inverted (18.9%) whereas mandibular teeth were more commonly inclined (32.7%).

Concerning the relationship between the follicle of the supernumerary tooth and adjacent teeth (Table 2), whether supernumerary or not, there were no significant differences between regions for the maxilla (*p* = 0.062) and for the mandible (*p* = 0.596), although there was an overall difference (*p* = 0.024). Considering the total sample, among supernumerary teeth in the incisor region, 56 (56%) had no contact, 19 (19%) were in contact, and 25 (25%) were fused. In the canine region, 21 (61.8%) had no contact, 9 (26.5%) were in contact, and 4 (11.8%) were fused. Among premolars, 130 (62.2%) had no contact, 58 (27.8%) were in contact, and 21 (10%) were fused. In the molar region, 43 (67.2%) had no contact, 12 (18.8%) were in contact, and 9 (14.1%) were fused.

The detection frequency of the gubernacular canal in the evaluated teeth, according to the dental arch and region is shown in Table 3. In the maxilla, the detection rate of the gubernacular canal varied between 31.2% and 45.8%, without statistical significance among regions (*p* = 0.326). However, in the mandible, the detection rate varied from 15.0% (molars) to 58.6% (premolars), with a statistically significant difference among regions (*p* = 0.001). Likewise, the overall detection rate of the gubernacular canal, regardless of dental arch, varied from 31.0% (incisors) to 56% (premolars), with a statistically significant difference among regions (*p* < 0.001).

**Fig. 2:**
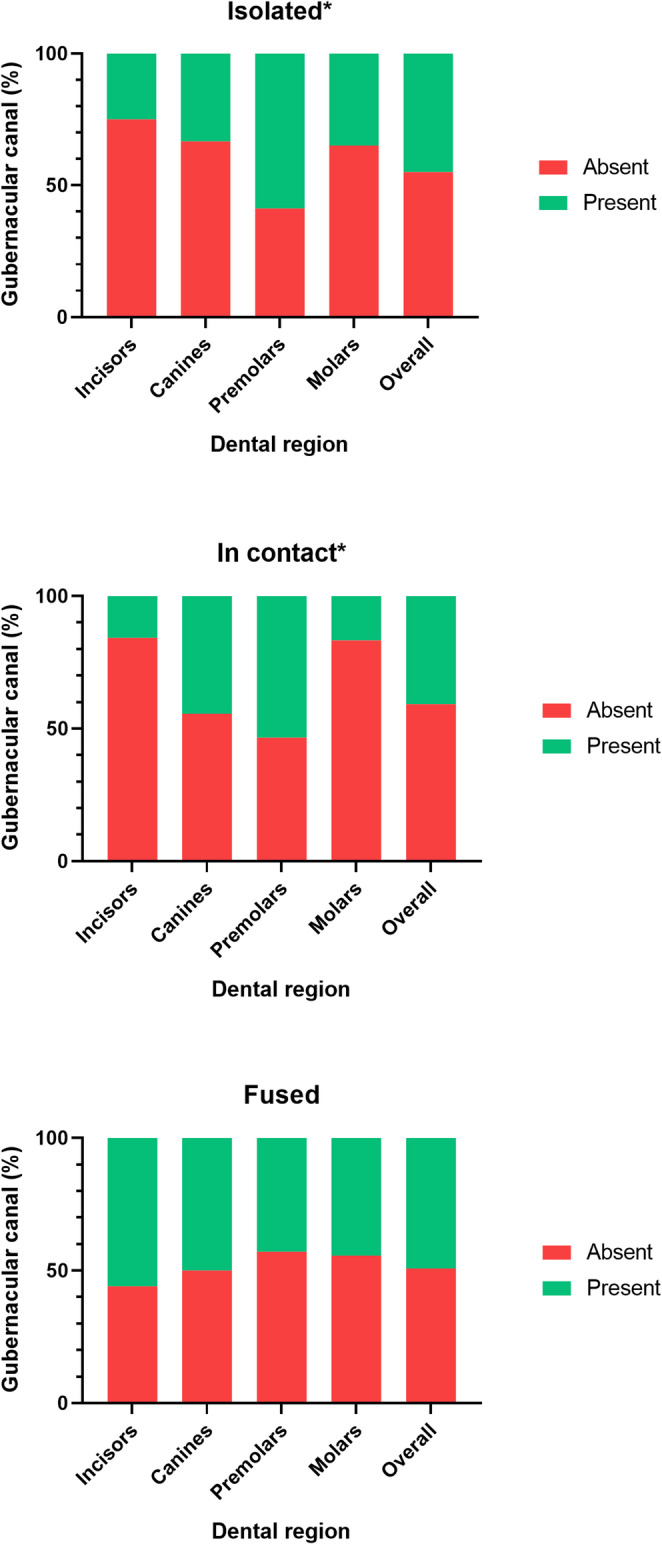
Stacked bar charts showing the relative frequency (%) of gubernacular canal detection in different dental groups, according to the classification of the relationship with adjacent teeth follicles: isolated (top), in contact (middle), and fused (bottom). An asterisk indicates statistically significant difference (*p*<0.05) according to the Chi-square test

As shown in Fig. 2, in cases classified as isolated (top chart), the absence of the gubernacular canal was more frequent in the anterior regions (incisors and canines), whereas its presence was more commonly observed in premolars (*p* < 0.001). In the in contact group (middle chart), the absence of the canal predominated, especially in the external groups (incisors and molars), while canines and premolars showed relatively higher percentages of presence (*p* = 0.008). Among supernumerary teeth with fused follicles (bottom chart), there was a balance between the presence and absence of the gubernacular canal among regions (*p* = 0.830).

**Fig. 3:**
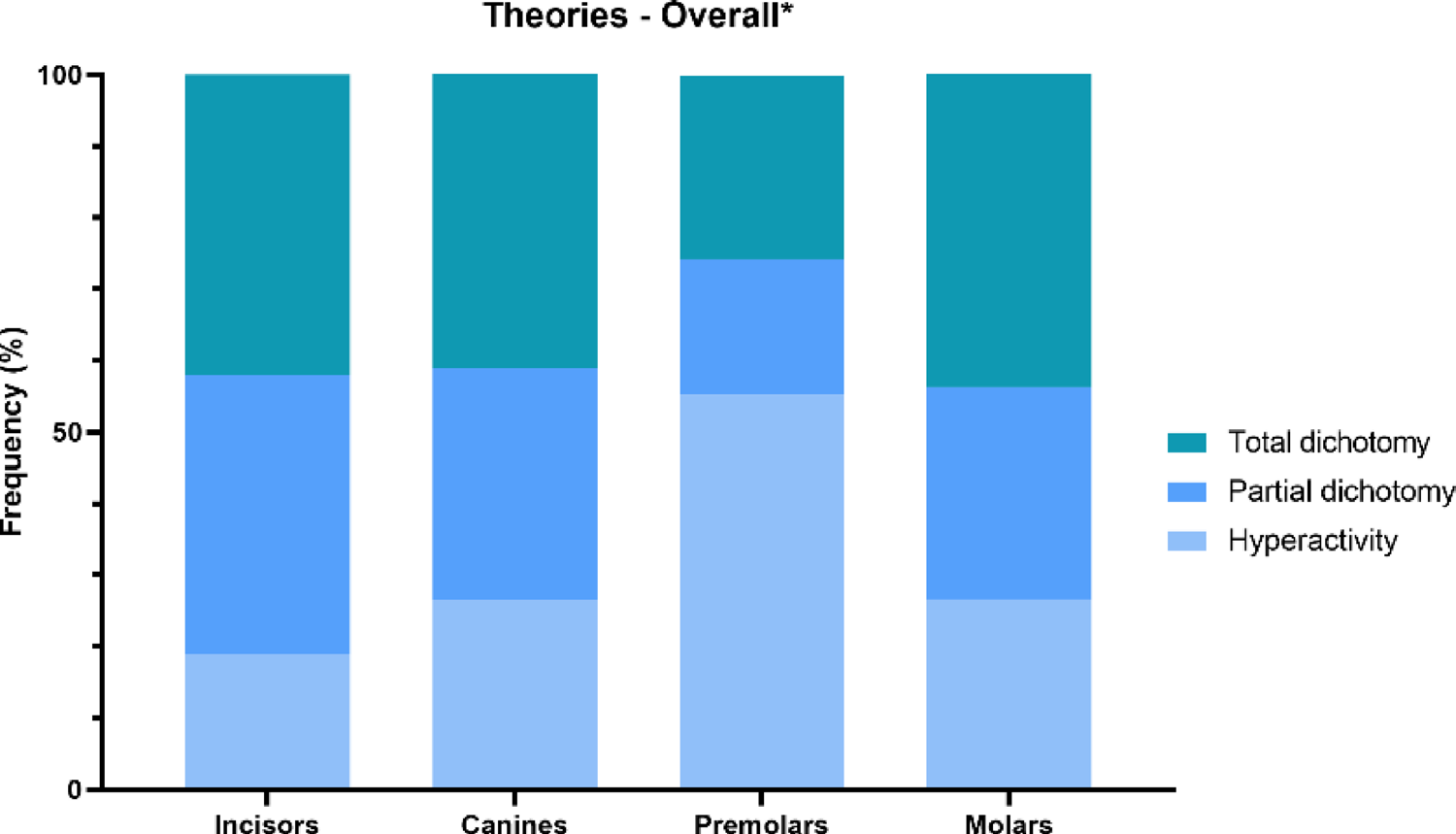
Bar chart showing the relative frequency (%) of patterns consistent with each suggested developmental theory in the different dental groups, considering the phases of dichotomy (total or partial). An asterisk indicates statistically significant difference (*p*<0.05) according to the Chi-square test

**Fig. 4:**
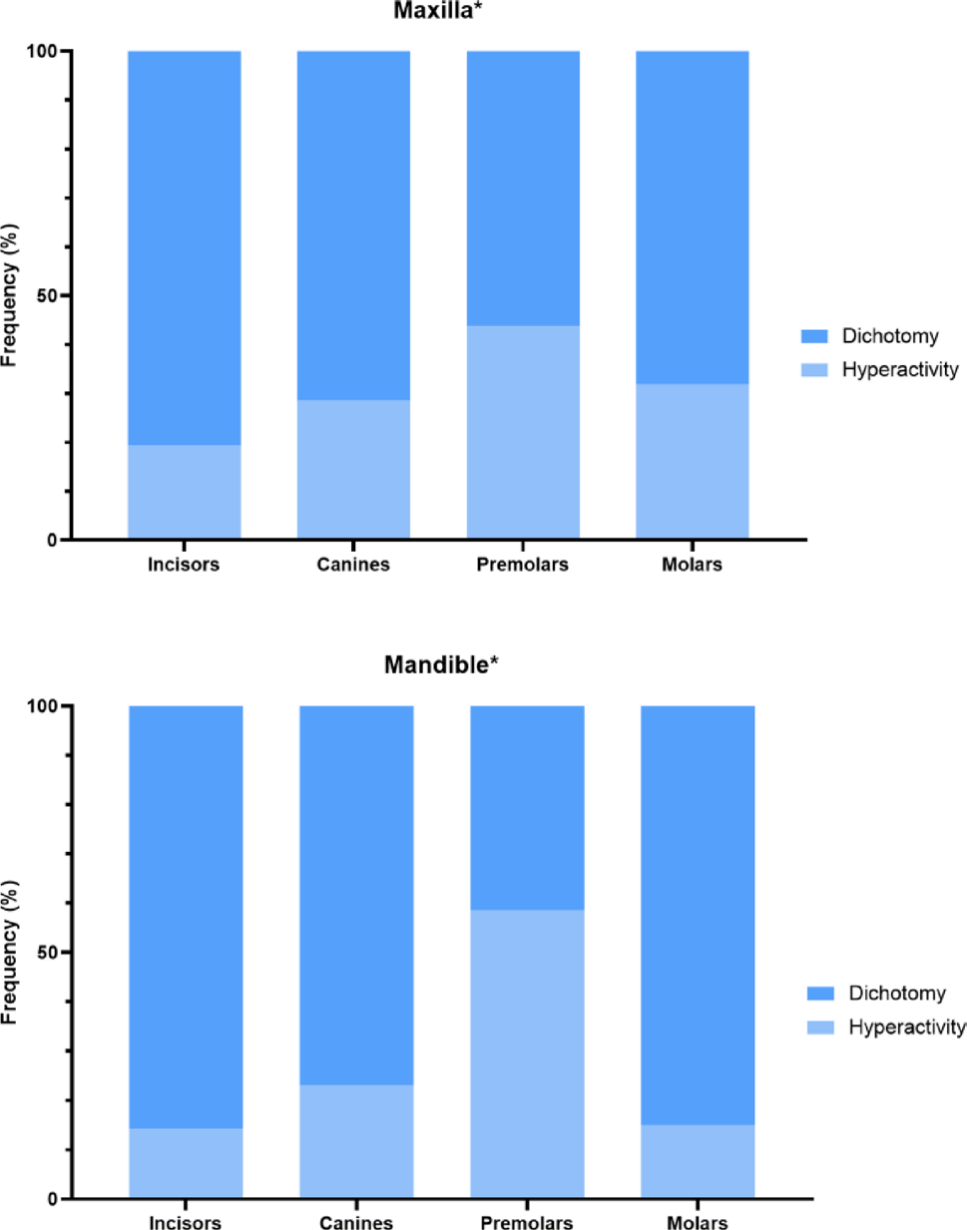
Bar charts showing the relative frequency (%) of occurrence of patterns consistent with each suggested developmental theory in the different dental groups for the maxilla (top) and the mandible (bottom), considering the dichotomy theory and the dental lamina hyperactivity theory. An asterisk indicates statistically significant difference (*p*<0.05) according to the Chi-square test

Figures 3 and 4 shows the frequency of the theories that could explain supernumerary teeth development in the different regions of the jaws. Overall, there was a statistically significant difference among regions (*p* < 0.001), suggesting a higher frequency of dental lamina hyperactivity in premolars (55%) and a proportional distribution between total and partial dichotomies across all regions (Fig. 3). When analyzing dental arches separately (Fig. 4), there was a statistically significant difference among regions for the maxilla (*p* = 0.023) and for the mandible (*p* < 0.001), suggesting that although the dichotomy theory predominated in all regions, teeth in the premolar region could be related more often to the hyperactivity theory (Fig. 4).

**Table 1:** Absolute (n) and relative (%) frequency of the descriptive variables of supernumerary teeth, according to the dental arch

Descriptive variables		Maxilla *N* (%)	Mandible *N* (%)	Total *N* (%)	*p*-value*
Region	Incisors	93 (45.1)	7 (3.5)	100 (24.5)	**<0.001**
	Canines	21 (10.2)	13 (6.4)	34 (8.3)	
	Premolars	48 (23.3)	162 (80.2)	210 (51.5)	
	Molars	44 (21.4)	20 (9.9)	64 (15.7)	
Development	Crypt	4 (1.9)	4 (2)	8 (2)	**0.027**
	Crown	68 (33)	66 (32.7)	134 (32.8)	
	< ½ of the root	23 (11.2)	22 (10.9)	45 (11)	
	> ½ of the root	22 (10.7)	45 (22.3)	67 (16.4)	
	Complete	79 (38.3)	54 (26.7)	133 (32.6)	
	Indeterminate	10 (4.9)	11 (5.4)	21 (5.1)	
Morphology	Normal	71 (34.5)	154 (76.2)	224 (55.1)	**<0.001**
	Altered	119 (57.8)	33 (16.3)	152 (37.3)	
	Odontoma	10 (4.9)	9 (4.5)	19 (4.7)	
	Indeterminate	6 (2.9)	6 (3)	12 (2.9)	
Inclination	Vertical	99 (48.1)	111 (55)	210 (51.5)	**<0.001**
	Inclined	38 (18.4)	66 (32.7)	104 (25.5)	
	Horizontal	19 (9.2)	15 (7.4)	34 (8.3)	
	Inverted	39 (18.9)	1 (0.5)	40 (9.8)	
	Indeterminate	11 (5.3)	9 (4.5)	20 (4.9)	

*According to the chi-square test. Values in bold indicate statistically significant differences (*p* < 0.05)

**Table 2:** Absolute (n) and relative (%) frequency of the relationship between the dental follicle and adjacent developing teeth, according to the arch and dental region

Arch	Region	Dental follicle N (%)			p-value*
		Isolated	In contact	Fused	
Maxilla	Incisors	53 (57)	16 (17.2)	24 (25.8)	0.062
	Canines	14 (66.7)	6 (28.6)	1 (4.8)	
	Premolars	34 (70.8)	8 (16.7)	6 (12.5)	
	Molars	34 (77.3)	4 (9.1)	6 (13.6)	
Mandible	Incisors	3 (42.9)	3 (42.9)	1 (14.3)	0.596
	Canines	7 (53.8)	3 (23.1)	3 (23.1)	
	Premolars	97 (59.9)	50 (30.9)	15 (9.3)	
	Molars	9 (45)	8 (40)	3 (15)	
Total	Incisors	56 (56)	19 (19)	25 (25)	**0.024**
	Canines	21 (61.8)	9 (26.5)	4 (11.8)	
	Premolars	130 (62.2)	58 (27.8)	21 (10)	
	Molars	43 (67.2)	12 (18.8)	9 (14.1)	

*According to the chi-square test. Values in bold indicate statistically significant differences (*p* < 0.05)

**Table 3:** Absolute (n) and relative (%) frequency of gubernacular canal detection, according to the arch and dental region

Arch	Region	Gubernacular canal N (%)		p-value*
		Absent	Present	
Maxilla	Incisors	64 (68.8)	29 (31.2)	0.326
	Canines	12 (57.1)	9 (42.9)	
	Premolars	26 (54.2)	22 (45.8)	
	Molars	26 (59.1)	18 (40.9)	
Mandible	Incisors	5 (71.4)	2 (28.6)	**0.001**
	Canines	9 (69.2)	4 (30.8)	
	Premolars	67 (41.4)	95 (58.6)	
	Molars	17 (85)	3 (15)	
Total	Incisors	69 (69)	31 (31)	**<0.001**
	Canines	21 (61.8)	13 (38.2)	
	Premolars	92 (44)	117 (56)	
	Molars	43 (67.2)	21 (32.8)	

*According to the chi-square test. Values in bold indicate statistically significant differences (*p* < 0.05)

## Discussion

The present study aimed to evaluate the characteristics of the dental follicle and the detection rate of the gubernacular canal in supernumerary teeth from different regions of the dental arches, using CBCT scans in non-syndromic patients, in order to seek evidence that could support theories of the formation of these teeth. Based on this approach, the findings allow discussion not only of the frequency and presentation patterns of the structures analyzed but also of their possible relationship with developmental theories of supernumerary teeth, among other hypotheses that will be detailed throughout the discussion.

According to the study by Coelho et al., 2011 [11] on the prevalence and distribution of supernumerary teeth in a pediatric population, mesiodens, supernumerary teeth located in the upper midline, constitute the most frequent type in the maxilla, while the premolar region is the most common for mandibular supernumeraries. The results of that study corroborate our findings, where the majority of maxillary supernumeraries were located in the incisor region (45.1%) and the majority of mandibular supernumeraries were in the premolar region (80.2%). Additionally, the stage of formation of the analyzed supernumeraries also showed differences between maxilla and mandible, with a higher prevalence of fully formed teeth in the maxilla.

Regarding morphology, according to Singh et al., 2014 [12], most supernumeraries have conical morphology, followed by tuberculate and supplemental. Similarly, in our study, altered morphology was the most frequent in the maxilla, as the most common maxillary supernumeraries were mesiodens, which usually present a conical shape [13]. Observing the findings related to inclination, there was a predominance of supernumeraries positioned vertically, in both maxilla and mandible, followed by inclined. It is also noteworthy that the vast majority of supernumeraries with inverted positions were located in the maxilla, which can be explained by the large number of mesiodens, teeth that are frequently positioned this way, as observed in the study by Mukhopadhyay et al., 2011 [14], in which 30.8% of mesiodens were classified as inverted.

Regarding the dental follicle of adjacent teeth, no significant difference was observed between the maxilla and mandible individually; however, there was a difference when considering the total sample. Fused follicles were more common in supernumeraries located in the incisor region, contact follicles were more common in the canine and premolar regions, and isolated follicles in the molar region. According to Nishina et al., 2024 [3], the specific composite structure of the gubernacular canal and dental follicle may reflect the development of two or three teeth from a single dental lamina, highlighting the relevance of follicles in the dynamics of tooth formation. This observation reinforces the idea that understanding the relationship between follicles is fundamental to understanding the mechanisms of formation and development of supernumerary teeth, as well as their eruption process and relationship with the gubernacular canal. However, it is important to note that this finding [3] comes from an exclusively syndromic population (cleidocranial dysplasia), which limits its generalization and underscores the need for additional investigations in non-syndromic patients, as in the present study.

Values for canal detection ranged from 15.0% to 58.6%, being lowest for supernumeraries located in the mandibular molar region and highest in the mandibular premolar region. The detection rate of the gubernacular canal is highly variable according to the existing literature. Gaêta-Araujo et al., 2019 [4] reported a detection rate of approximately 90.6% in normally developing teeth. Regarding supernumeraries, Zengin et al., 2023 [10] reported a detection rate of 77.2% for impacted supernumeraries, whereas in this study, considering supernumeraries in the mandibular premolar region, the detection rate of the gubernacular canal was nearly 59.0%, and overall, considering premolar supernumeraries in both maxilla and mandible, it was 56.0%. Thus, several studies have shown that detection rates of the gubernacular canal in supernumerary teeth are consistently lower compared to normally developing teeth, reinforcing the fundamental role of the dental follicle and its relationship with the gubernacular canal in the eruption process of teeth.

In addition to the role of the follicle in the eruption process, analyzing the proximity between adjacent follicles and the presence or absence of the gubernacular canal allows formulation of hypotheses regarding their possible association with the developmental theories of supernumerary teeth. Analyzing isolated teeth, the presence of the gubernacular canal was higher in supernumeraries located in the premolar region; in teeth with contact between adjacent follicles, detection was higher in supernumeraries in the premolar region, and in fused teeth, there was no difference in canal presence proportions across different regions of the arch. It is also noteworthy that only isolated and contact follicles showed statistically significant differences.

From a careful analysis of the findings regarding the relationship between dental follicles and the gubernacular canal, hypotheses were raised regarding their possible association with the main developmental theories of supernumerary teeth: the dichotomy theory and dental lamina hyperactivity. Isolated follicles without a gubernacular canal suggest total dichotomy, meaning a normally developing tooth underwent germ division that generated the supernumerary, which has an isolated follicle with no relation to the tooth it originated from, so the dichotomy occurred fully. Conversely, isolated follicles with a gubernacular canal suggest the theory of dental lamina hyperactivity, meaning that during odontogenesis, excessive lamina activity results in the formation of new independent tooth germs, in addition to the normal ones. These, therefore, as independent teeth, have their own gubernacular canal.

Supernumeraries with contact follicles without a gubernacular canal suggest partial dichotomy, as they do not have their own canal, coming from dichotomy but still maintaining contact with the follicle of the originating tooth. Teeth with contact follicles and a gubernacular canal suggest dental lamina hyperactivity and are considered independent teeth, as they have their own canal. For fused follicles, those without a canal suggest partial dichotomy, while fused follicles with a canal may originate from lamina hyperactivity if the canal is from it, or partial dichotomy if the canal is related to the other tooth. It is noteworthy that the hypotheses developed in this study have not yet been identified in the current literature, and thus this work seeks to expand knowledge of the potential relationship between these structures and supernumerary tooth development theories.

The distribution of the theories described above explaining the possible development of the supernumerary teeth was further explored according to the arch and the region (Figs. 3 and 4). It could be observed that the proportions of dichotomy and hyperactivity theories explaining the development of these teeth are similar in the maxilla and mandible. Notwithstanding, data does not directly observe one development theory over another but rather suggests the relationship with the different regions of the dental arches. Overall, there is a general predominance of dichotomy, especially in the maxilla, while for mandibular premolars region, supernumerary teeth could be mostly derived from lamina hyperactivity. A possible explanation for this, as suggested by Khalaf et al., 2018 [15], is that supernumerary premolar teeth may belong to a postpermanent dentition (third series), developing from extensions of the dental lamina. Unlike in other regions, and especially in the mandible, supernumerary teeth in the premolar region tend to have supplemental morphology [15]. Importantly, the division between partial and total dichotomy is also shown, with both exhibiting a balanced distribution across all regions of the dental arch. The different phases of dichotomy can be explained by a true incomplete process of the division of the dental follicle but also resemble the timely characteristic of the process at the time the patient was scanned.

The limitations of this study are related to inherent constraints of any cross-sectional study, as the data collected corresponds to imaging features at a specific moment and does not reflect the sequence of events during tooth eruption, nor do they allow identification of dental follicle or gubernacular canal variations over time. In this sense, some fully formed teeth were observed without a visible canal, which may suggest that formed teeth could be associated with canal resorption due to prolonged retention in the arch. However, this association was not directly evaluated in this study, and future investigations are necessary to explore this phenomenon in more detail. Additionally, a control group of normal or non-supernumerary teeth was not included in the present study, as it is expected that normal teeth have the presence of the gubernacular canal, except in abnormal eruption process. Furthermore, histological analysis for investigating developmental origins was not viable due to the imaging-based and retrospective nature of this study. Therefore, the findings should be interpreted within the context of CBCT assessments. In addition to being a cross-sectional study, another limitation is that the entire image database comes from a single center, i.e., restricted to a specific and similar-profile population, which may limit generalization of results to other contexts and populations, requiring further investigations.

On the other hand, the present study presents important positive aspects that contribute to the relevance of the findings. The sample analyzed was relatively large, totaling 408 teeth, a number defined based on a sample size calculation, ensuring greater precision and representativeness of the results. Unlike previous studies, this research explores the possible association of gubernacular canal characteristics with main developmental theories of supernumerary teeth, providing an innovative and well-founded approach. Additionally, the patients included in the study were non-syndromic, unlike the study by Nishina et al., 2024 [3], which ensures that the findings reflect typical characteristics of the general population, without interference from systemic conditions or syndromes that could bias the results.

## Conclusion

Thus, it can be inferred that, depending on the region analyzed, different types of follicular relationships predominate, as well as varying detection rates of the gubernacular canal, suggesting that distinct regions could be associated with different developmental theories of supernumerary teeth. Isolated follicles are predominant in all regions, followed by in contact and fused follicles, except in the incisor region, where there is a predominance of fused follicles over in contact. Regarding the detection rate of the gubernacular canal, its presence is more frequent than its absence only in the premolar region. In all other regions, absence of the gubernacular canal is predominant. Furthermore, most incisors, canines, and molars could be associated with the dichotomy theory, whereas in the premolar region, the most frequent suggested theory was that of dental lamina hyperactivity.

These findings provide clinically relevant insights for the understanding of CBCT features of supernumerary teeth, the appearance of their corresponding dental follicle and gubernacular canal. Anticipating these features may assist clinicians in distinguishing these teeth from other radiographically similar structures and in foreseeing their eruption behavior.

## Data Availability

The data can be accessed on reasonable request to the corresponding author.

## References

[1] Cunha D, Ferreira A, Fumes AC, et al. Gubernacular Cord and Canal - Does these anatomical structures play a role in dental eruption? Revista Sul-Brasileira de Odontologia. 2013;10:167–71.

[2] Cahill DR, Marks SC. Tooth eruption: evidence for the central role of the dental follicle. J Oral Pathol Med. 1980;9:189–200. 10.1111/j.1600-0714.1980.tb00377.x. 10.1111/j.1600-0714.1980.tb00377.x 6777476

[3] Nishina S, Oda M, Nishida I, et al. Imaging characteristics of gubernaculum tracts in patients with cleidocranial dysplasia: a computed tomography study. Oral Surg Oral Med Oral Pathol Oral Radiol. 2024;138:556–64. 10.1016/j.oooo.2024.04.106. 38839481 10.1016/j.oooo.2024.04.106

[4] Gaêta-Araujo H, Da Silva MB, Tirapelli C, et al. Detection of the gubernacular canal and its attachment to the dental follicle may indicate an abnormal eruption status. Angle Orthod. 2019;89:781–7. 10.2319/090518-651.1. 30855183 10.2319/090518-651.1 PMC8111843

[5] Oda M, Nishida I, Miyamoto I, et al. Significance and usefulness of imaging characteristics of gubernaculum tracts for the diagnosis of odontogenic tumors or cysts. PLoS ONE. 2018;13. 10.1371/journal.pone.0199285. 10.1371/journal.pone.0199285 PMC6034793 29979687

[6] Oda M, Miyamoto I, Nishida I, et al. A spatial association between odontomas and the gubernaculum tracts. Oral Surg Oral Med Oral Pathol Oral Radiol. 2016;121:91–5. 10.1016/j.oooo.2015.10.014. 26679362 10.1016/j.oooo.2015.10.014

[7] Liu S, Lin Z, Wen S, et al. Epidemiological and CBCT characterizations of odontomas: A retrospective study of 87,590 subjects. Oral Dis. 2024;30:4585–97. 10.1111/odi.14845. 38129744 10.1111/odi.14845

[8] Anthonappa RP, King NM, Rabie ABM. Aetiology of supernumerary teeth: A literature review. Eur Archives Pediatr Dentistry. 2013;14:279–88. 10.1007/s40368-013-0082-z 24068489

[9] Kaplan FA, Bilgir E, Bayrakdar İŞ, Kılıç MÇ (2020) Evaluation of gubernacular tract with cone beam computed tomography in impacted supernumerary teeth.

[10] Zengin AZ, Rizeli L, Sumer AP. Detection and characteristics of the gubernacular tract in supernumerary teeth on cone beam computed tomography. Oral Radiol. 2023;39:292–300. 10.1007/s11282-022-00636-9. 35907117 10.1007/s11282-022-00636-9

[11] Coelho A, Macho V, Andrade D, et al. Prevalence and distribution of supernumerary teeth in a pediatric population - a radiographic study. Revista Portuguesa de Estomatologia Med Dentaria e Cirurgia Maxilofacial. 2011;52:189–92. 10.1016/j.rpemd.2011.09.005.

[12] Singh VP, Sharma A, Sharma S. Supernumerary teeth in nepalese children. Sci World J 2014. 2014. 10.1155/2014/215396. 10.1155/2014/215396 PMC4258912 25506609

[13] Sulabha AN, Sameer C. Association of Mesiodentes and Dens Invaginatus in a Child: A Rare Entity. Case Rep Dent. 2012;2012:1–4. 10.1155/2012/198032. 10.1155/2012/198032 PMC3502804 23198162

[14] Mukhopadhyay S. Mesiodens: A clinical and radiographic study in children. J Indian Soc Pedod Prev Dentistry. 2011;29:34–8. 10.4103/0970-4388.79928. 10.4103/0970-4388.79928 21521916

[15] Khalaf K, Al Shehadat S, Murray CA (2018) A Review of Supernumerary Teeth in the Premolar Region. Int. J. Dent. 2018. 10.1155/2018/6289047 PMC6304893 30631362

